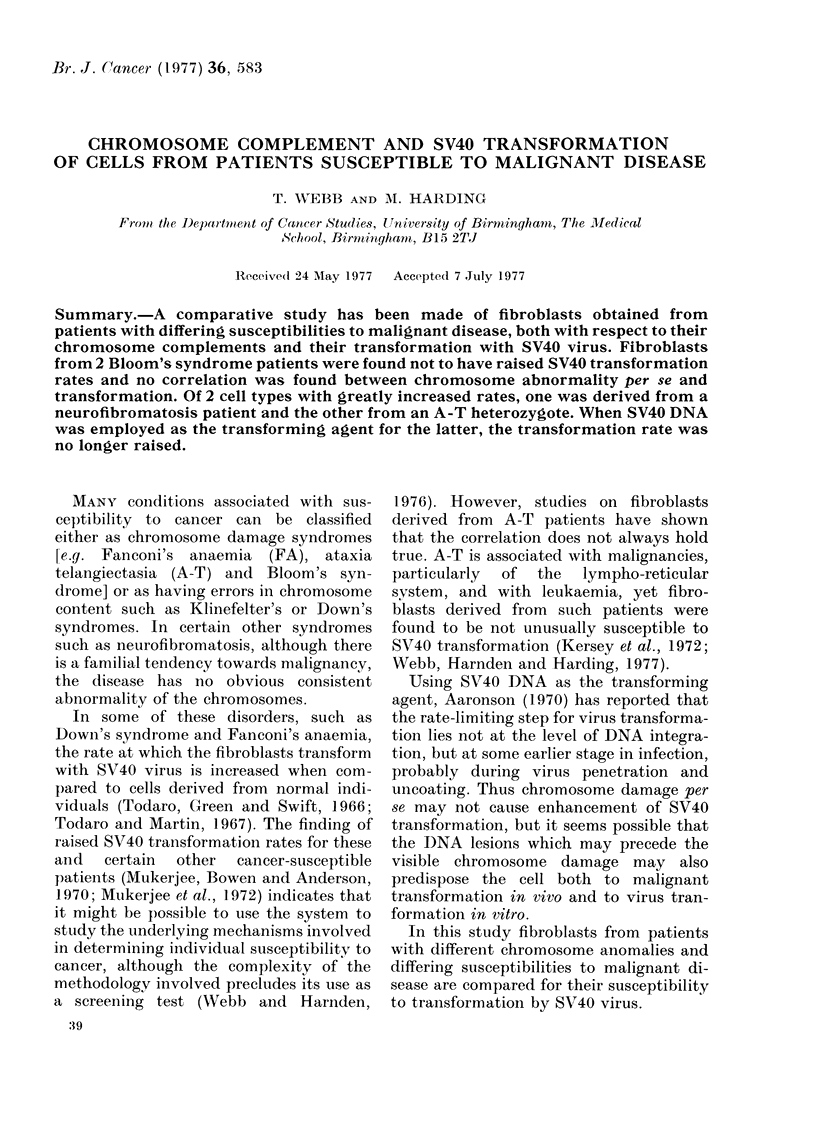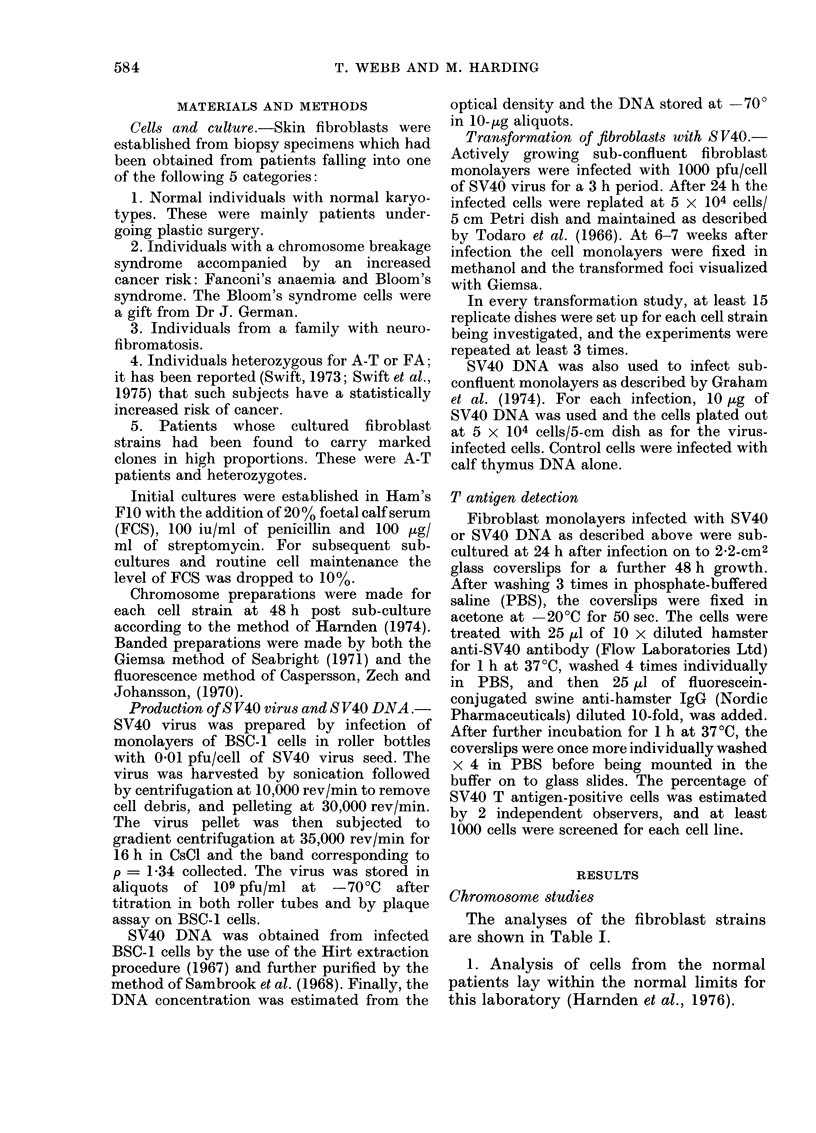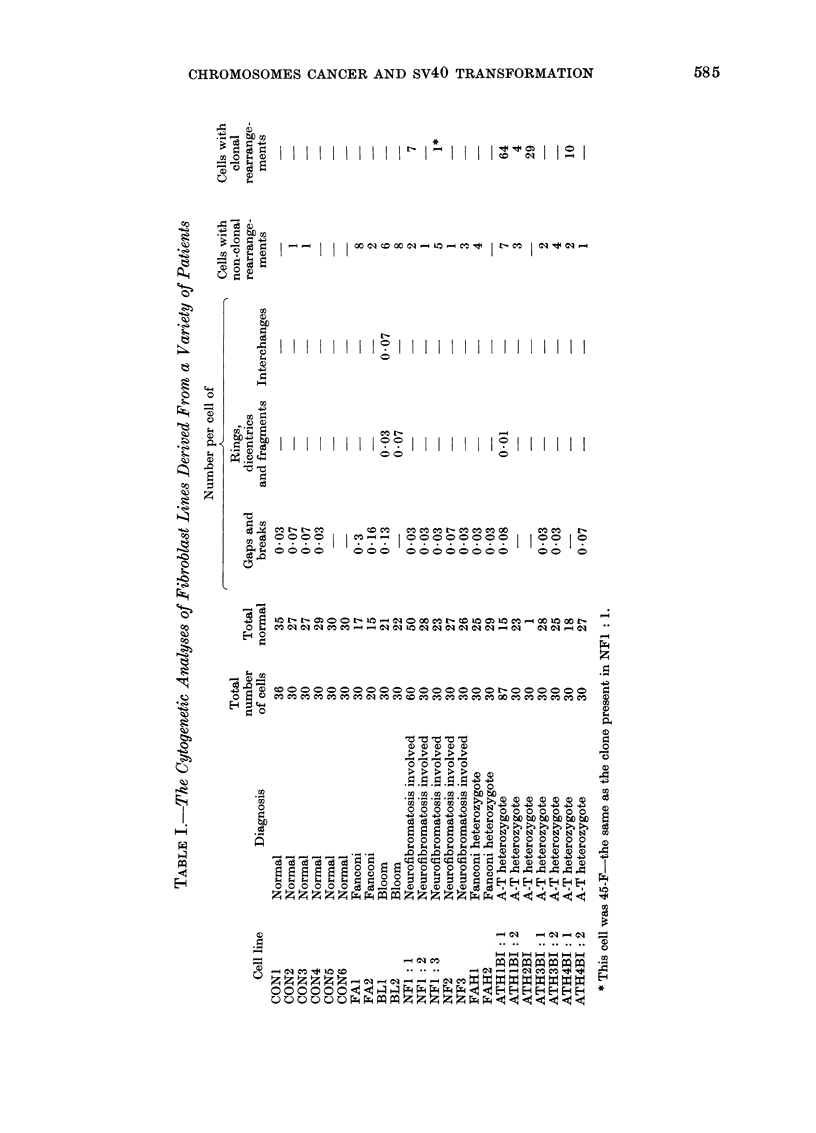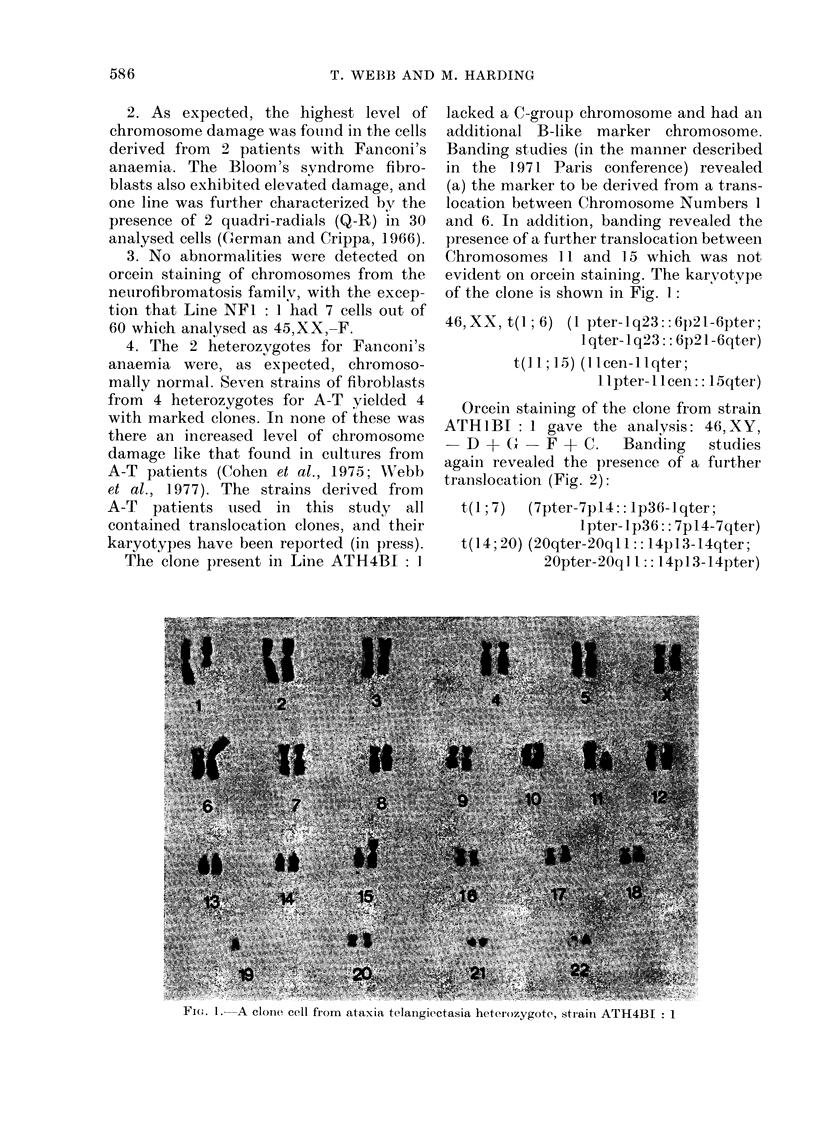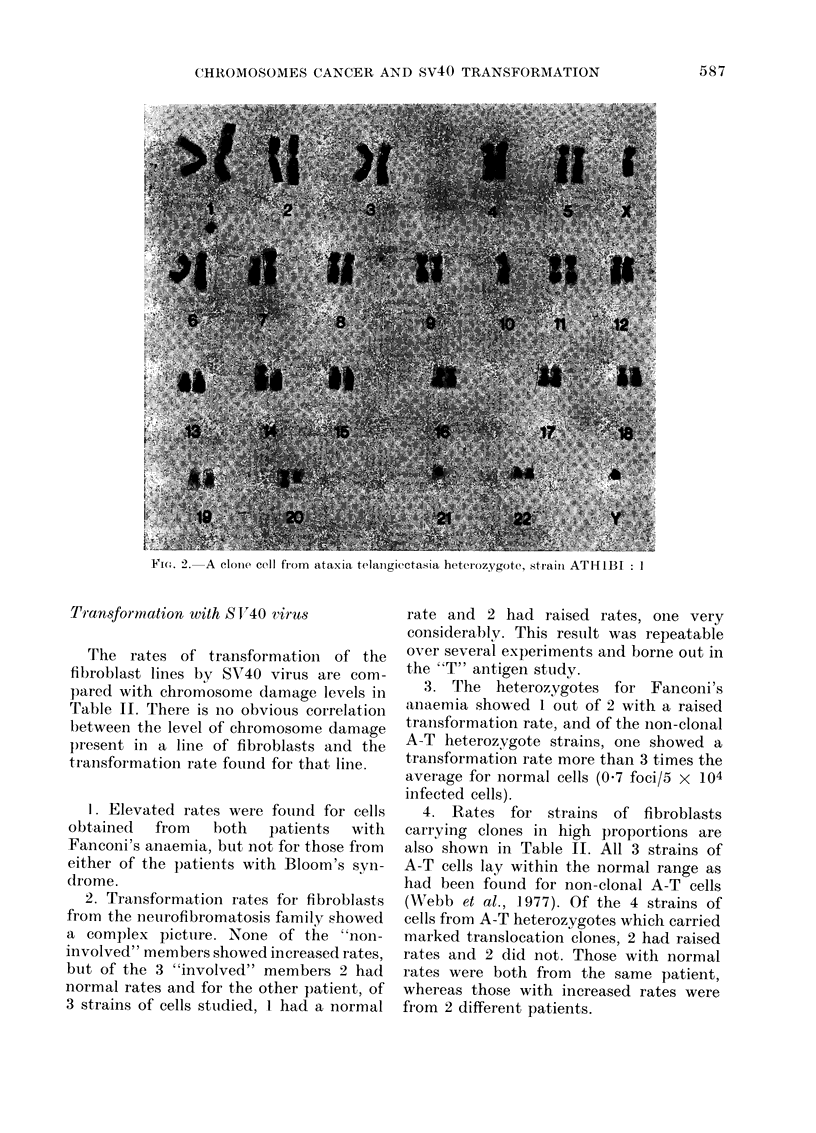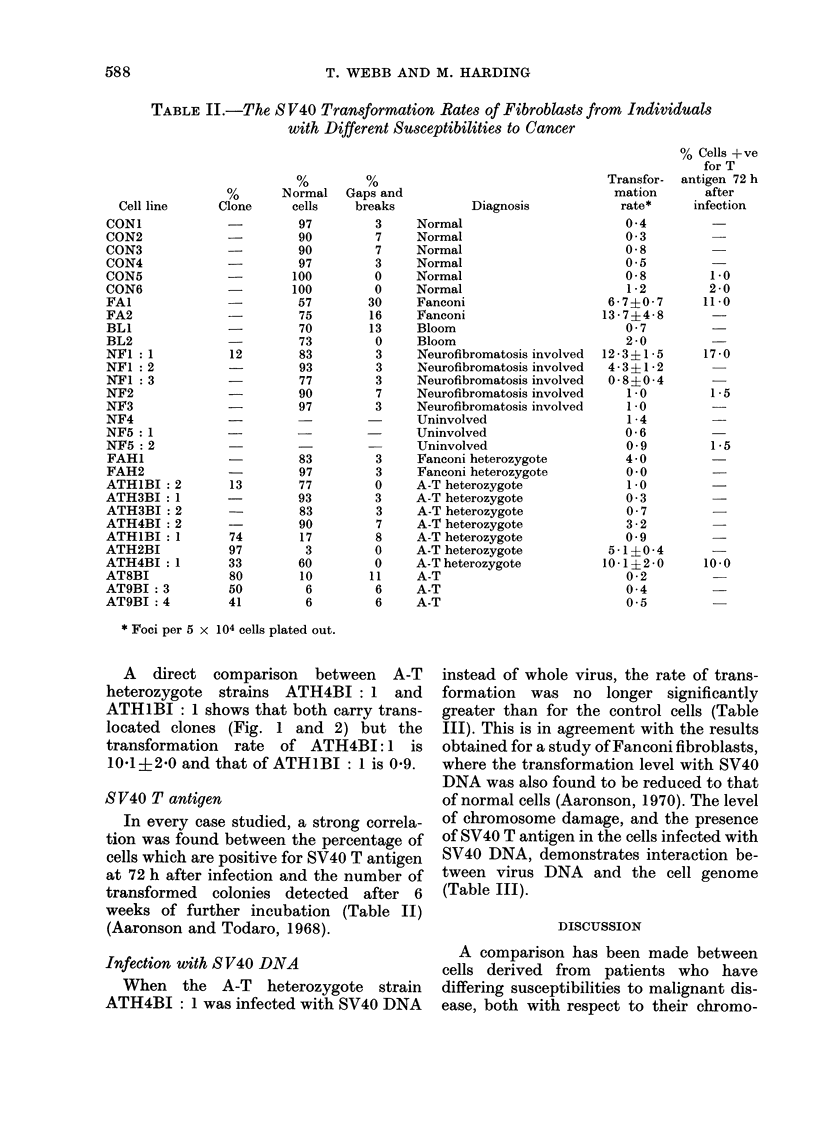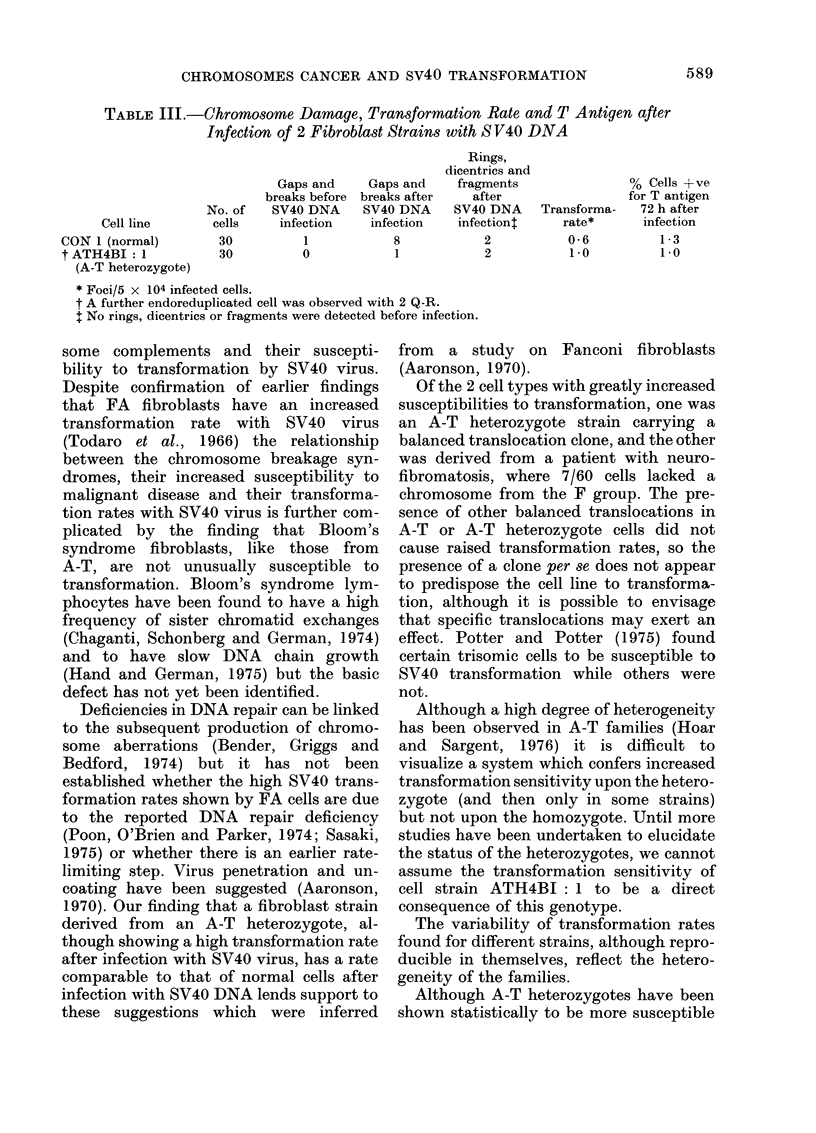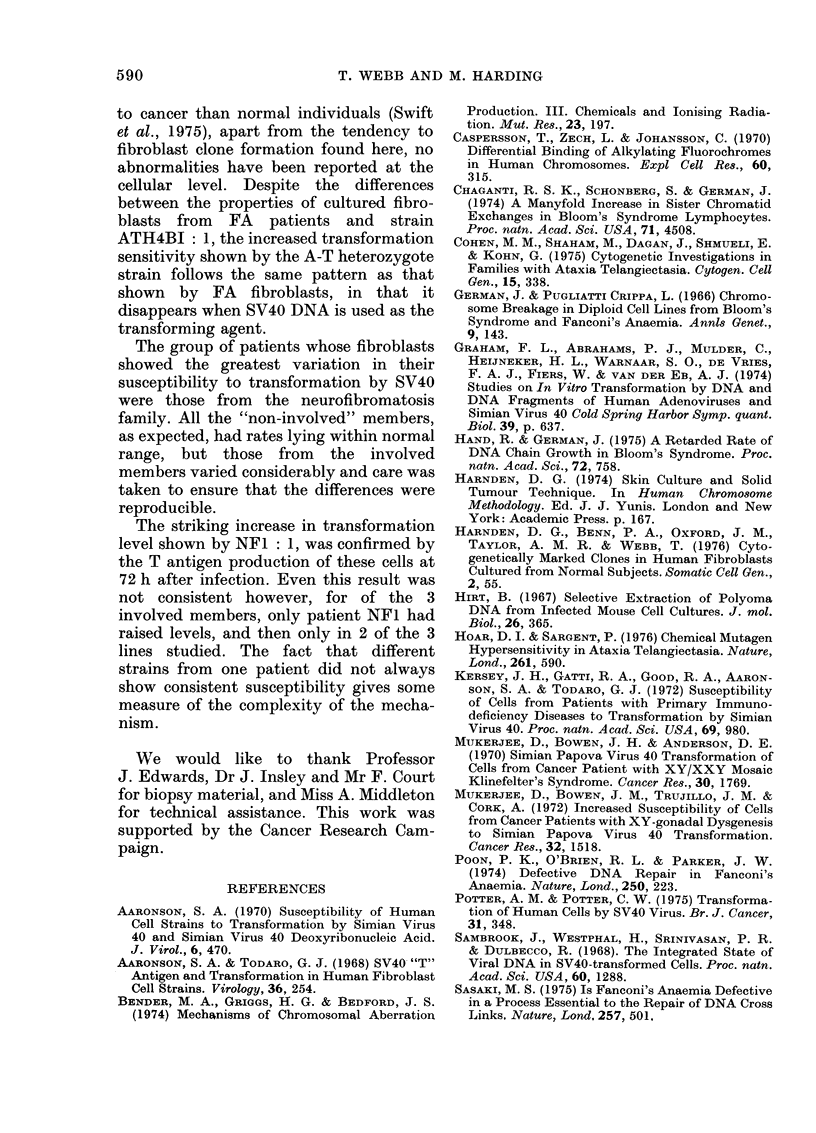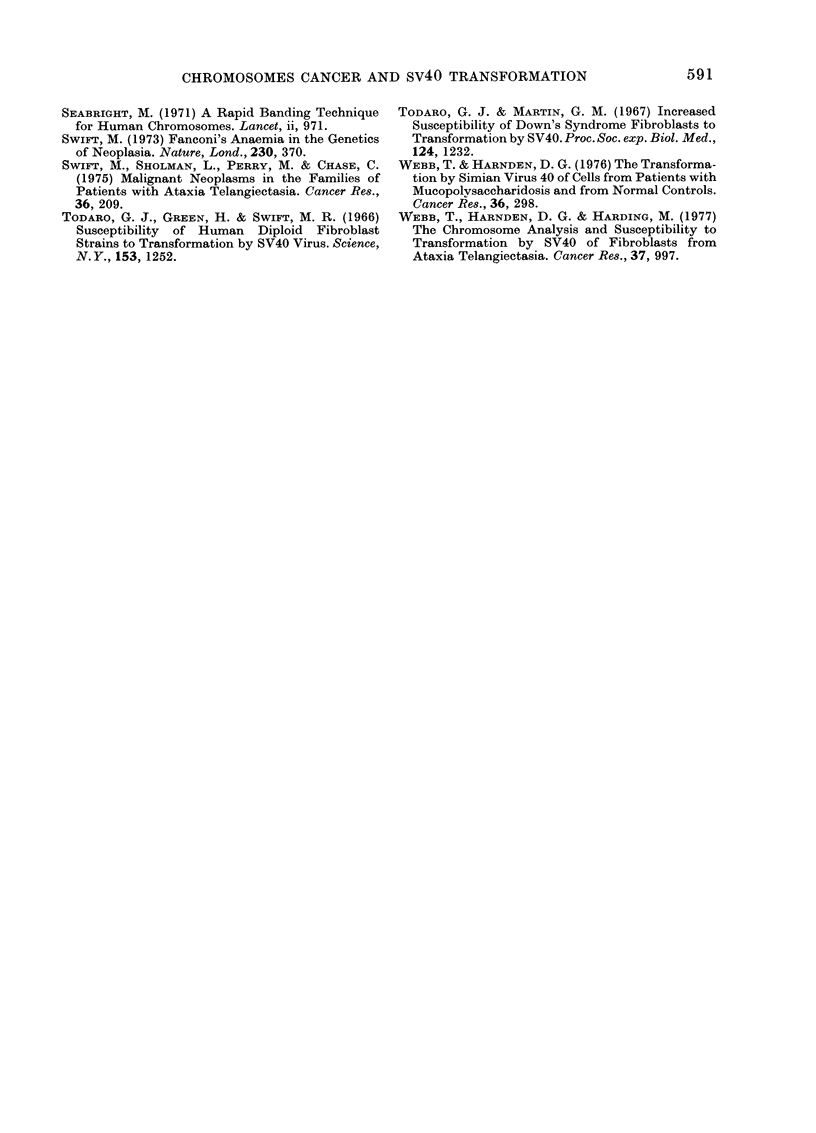# Chromosome complement and SV40 transformation of cells from patients susceptible to malignant disease.

**DOI:** 10.1038/bjc.1977.235

**Published:** 1977-11

**Authors:** T. Webb, M. Harding

## Abstract

**Images:**


*Br. J. Cancer* (1977) **36**, 583

### CHROMOSOME COMPLEMENT AND SV40 TRANSFORMATION OF CELLS FROM PATIENTS SUSCEPTIBLE TO MALIGNANT DISEASE

T. WEBB AND M. HARDING

*From the Department of Cancer Studies, University of Birmingham, The Medical School, Birmingham, B15 2TJ*

Received 24 May 1977 Accepted 7 July 1977

**Summary.—A comparative study has been made of fibroblasts obtained from patients with differing susceptibilities to malignant disease, both with respect to their chromosome complements and their transformation with SV40 virus. Fibroblasts from 2 Bloom's syndrome patients were found not to have raised SV40 transformation rates and no correlation was found between chromosome abnormality *per se* and transformation. Of 2 cell types with greatly increased rates, one was derived from a neurofibromatosis patient and the other from an A-T heterozygote. When SV40 DNA was employed as the transforming agent for the latter, the transformation rate was no longer raised.**

MANY conditions associated with susceptibility to cancer can be classified either as chromosome damage syndromes [*e.g.* Fanconi's anaemia (FA), ataxia telangiectasia (A-T) and Bloom's syndrome] or as having errors in chromosome content such as Klinefelter's or Down's syndromes. In certain other syndromes such as neurofibromatosis, although there is a familial tendency towards malignancy, the disease has no obvious consistent abnormality of the chromosomes.

In some of these disorders, such as Down's syndrome and Fanconi's anaemia, the rate at which the fibroblasts transform with SV40 virus is increased when compared to cells derived from normal individuals (Todaro, Green and Swift, 1966; Todaro and Martin, 1967). The finding of raised SV40 transformation rates for these and certain other cancer-susceptible patients (Mukerjee, Bowen and Anderson, 1970; Mukerjee *et al.*, 1972) indicates that it might be possible to use the system to study the underlying mechanisms involved in determining individual susceptibility to cancer, although the complexity of the methodology involved precludes its use as a screening test (Webb and Harnden, 1976). However, studies on fibroblasts derived from A-T patients have shown that the correlation does not always hold true. A-T is associated with malignancies, particularly of the lympho-reticular system, and with leukaemia, yet fibroblasts derived from such patients were found to be not unusually susceptible to SV40 transformation (Kersey *et al.*, 1972; Webb, Harnden and Harding, 1977).

Using SV40 DNA as the transforming agent, Aaronson (1970) has reported that the rate-limiting step for virus transformation lies not at the level of DNA integration, but at some earlier stage in infection, probably during virus penetration and uncoating. Thus chromosome damage *per se* may not cause enhancement of SV40 transformation, but it seems possible that the DNA lesions which may precede the visible chromosome damage may also predispose the cell both to malignant transformation *in vivo* and to virus tranformation *in vitro*.

In this study fibroblasts from patients with different chromosome anomalies and differing susceptibilities to malignant disease are compared for their susceptibility to transformation by SV40 virus.

### MATERIALS AND METHODS

*Cells and culture.*—Skin fibroblasts were established from biopsy specimens which had been obtained from patients falling into one of the following 5 categories:

1. Normal individuals with normal karyotypes. These were mainly patients undergoing plastic surgery.
2. Individuals with a chromosome breakage syndrome accompanied by an increased cancer risk: Fanconi's anaemia and Bloom's syndrome. The Bloom's syndrome cells were a gift from Dr J. German.
3. Individuals from a family with neurofibromatosis.
4. Individuals heterozygous for A-T or FA; it has been reported (Swift, 1973; Swift *et al.*, 1975) that such subjects have a statistically increased risk of cancer.
5. Patients whose cultured fibroblast strains had been found to carry marked clones in high proportions. These were A-T patients and heterozygotes.

Initial cultures were established in Ham's F10 with the addition of 20% foetal calf serum (FCS), 100 iu/ml of penicillin and 100 $\mu$g/ml of streptomycin. For subsequent subcultures and routine cell maintenance the level of FCS was dropped to 10%.

Chromosome preparations were made for each cell strain at 48 h post sub-culture according to the method of Harnden (1974). Banded preparations were made by both the Giemsa method of Seabright (1971) and the fluorescence method of Caspersson, Zech and Johansson, (1970).

*Production of SV40 virus and SV40 DNA.*—SV40 virus was prepared by infection of monolayers of BSC-1 cells in roller bottles with 0·01 pfu/cell of SV40 virus seed. The virus was harvested by sonication followed by centrifugation at 10,000 rev/min to remove cell debris, and pelleting at 30,000 rev/min. The virus pellet was then subjected to gradient centrifugation at 35,000 rev/min for 16 h in CsCl and the band corresponding to $\rho = 1{\cdot}34$ collected. The virus was stored in aliquots of $10^9$ pfu/ml at $-70$°C after titration in both roller tubes and by plaque assay on BSC-1 cells.

SV40 DNA was obtained from infected BSC-1 cells by the use of the Hirt extraction procedure (1967) and further purified by the method of Sambrook *et al.* (1968). Finally, the DNA concentration was estimated from the optical density and the DNA stored at $-70$° in 10-$\mu$g aliquots.

*Transformation of fibroblasts with SV40.*—Actively growing sub-confluent fibroblast monolayers were infected with 1000 pfu/cell of SV40 virus for a 3 h period. After 24 h the infected cells were replated at $5 \times 10^4$ cells/5 cm Petri dish and maintained as described by Todaro *et al.* (1966). At 6–7 weeks after infection the cell monolayers were fixed in methanol and the transformed foci visualized with Giemsa.

In every transformation study, at least 15 replicate dishes were set up for each cell strain being investigated, and the experiments were repeated at least 3 times.

SV40 DNA was also used to infect sub-confluent monolayers as described by Graham *et al.* (1974). For each infection, 10 $\mu$g of SV40 DNA was used and the cells plated out at $5 \times 10^4$ cells/5-cm dish as for the virus-infected cells. Control cells were infected with calf thymus DNA alone.

### *T antigen detection*

Fibroblast monolayers infected with SV40 or SV40 DNA as described above were sub-cultured at 24 h after infection on to 2·2-cm$^2$ glass coverslips for a further 48 h growth. After washing 3 times in phosphate-buffered saline (PBS), the coverslips were fixed in acetone at $-20$°C for 50 sec. The cells were treated with 25 $\mu$l of 10 $\times$ diluted hamster anti-SV40 antibody (Flow Laboratories Ltd) for 1 h at 37°C, washed 4 times individually in PBS, and then 25 $\mu$l of fluorescein-conjugated swine anti-hamster IgG (Nordic Pharmaceuticals) diluted 10-fold, was added. After further incubation for 1 h at 37°C, the coverslips were once more individually washed $\times$ 4 in PBS before being mounted in the buffer on to glass slides. The percentage of SV40 T antigen-positive cells was estimated by 2 independent observers, and at least 1000 cells were screened for each cell line.

### RESULTS

### *Chromosome studies*

The analyses of the fibroblast strains are shown in Table I.

1. Analysis of cells from the normal patients lay within the normal limits for this laboratory (Harnden *et al.*, 1976).

TABLE I.—*The Cytogenetic Analyses of Fibroblast Lines Derived From a Variety of Patients*

| Cell line | Diagnosis | Total number of cells | Total normal | Number per cell of: Gaps and breaks | Number per cell of: Rings, dicentrics and fragments | Number per cell of: Interchanges | Cells with non-clonal rearrangements | Cells with clonal rearrangements |
|---|---|---|---|---|---|---|---|---|
| CON1 | Normal | 36 | 35 | 0·03 | — | — | — | — |
| CON2 | Normal | 30 | 27 | 0·07 | — | — | 1 | — |
| CON3 | Normal | 30 | 27 | 0·07 | — | — | 1 | — |
| CON4 | Normal | 30 | 29 | 0·03 | — | — | — | — |
| CON5 | Normal | 30 | 30 | — | — | — | — | — |
| CON6 | Normal | 30 | 30 | — | — | — | — | — |
| FA1 | Fanconi | 30 | 17 | 0·3 | — | — | 8 | — |
| FA2 | Fanconi | 20 | 15 | 0·16 | — | — | 2 | — |
| BL1 | Bloom | 30 | 21 | 0·13 | 0·03 | 0·07 | 6 | — |
| BL2 | Bloom | 30 | 22 | — | 0·07 | — | 8 | — |
| NF1 : 1 | Neurofibromatosis involved | 60 | 50 | 0·03 | — | — | 2 | 7 |
| NF1 : 2 | Neurofibromatosis involved | 30 | 28 | 0·03 | — | — | 1 | — |
| NF1 : 3 | Neurofibromatosis involved | 30 | 23 | 0·03 | — | — | 5 | 1* |
| NF2 | Neurofibromatosis involved | 30 | 27 | 0·07 | — | — | 1 | — |
| NF3 | Neurofibromatosis involved | 30 | 26 | 0·03 | — | — | 3 | — |
| FAH1 | Fanconi heterozygote | 30 | 25 | 0·03 | — | — | 4 | — |
| FAH2 | Fanconi heterozygote | 30 | 29 | 0·03 | — | — | — | — |
| ATH1BI : 1 | A-T heterozygote | 87 | 15 | 0·08 | 0·01 | — | 7 | 64 |
| ATH1BI : 2 | A-T heterozygote | 30 | 23 | — | — | — | 3 | 4 |
| ATH2BI | A-T heterozygote | 30 | 1 | — | — | — | — | 29 |
| ATH3BI : 1 | A-T heterozygote | 30 | 28 | 0·03 | — | — | 2 | — |
| ATH3BI : 2 | A-T heterozygote | 30 | 25 | 0·03 | — | — | 4 | — |
| ATH4BI : 1 | A-T heterozygote | 30 | 18 | — | — | — | 2 | 10 |
| ATH4BI : 2 | A-T heterozygote | 30 | 27 | 0·07 | — | — | 1 | — |

* This cell was 45-F—the same as the clone present in NF1 : 1.

2. As expected, the highest level of chromosome damage was found in the cells derived from 2 patients with Fanconi's anaemia. The Bloom's syndrome fibroblasts also exhibited elevated damage, and one line was further characterized by the presence of 2 quadri-radials (Q-R) in 30 analysed cells (German and Crippa, 1966).

3. No abnormalities were detected on orcein staining of chromosomes from the neurofibromatosis family, with the exception that Line NF1 : 1 had 7 cells out of 60 which analysed as 45,XX,–F.

4. The 2 heterozygotes for Fanconi's anaemia were, as expected, chromosomally normal. Seven strains of fibroblasts from 4 heterozygotes for A-T yielded 4 with marked clones. In none of these was there an increased level of chromosome damage like that found in cultures from A-T patients (Cohen *et al.*, 1975; Webb *et al.*, 1977). The strains derived from A-T patients used in this study all contained translocation clones, and their karyotypes have been reported (in press).

The clone present in Line ATH4BI : 1 lacked a C-group chromosome and had an additional B-like marker chromosome. Banding studies (in the manner described in the 1971 Paris conference) revealed (a) the marker to be derived from a translocation between Chromosome Numbers 1 and 6. In addition, banding revealed the presence of a further translocation between Chromosomes 11 and 15 which was not evident on orcein staining. The karyotype of the clone is shown in Fig. 1:

46,XX, t(1;6) (1 pter-1q23::6p21-6pter;

1qter-1q23::6p21-6qter)

t(11;15) (11cen-11qter;

11pter-11cen::15qter)

Orcein staining of the clone from strain ATH1BI : 1 gave the analysis: 46,XY, – D + G – F + C. Banding studies again revealed the presence of a further translocation (Fig. 2):

t(1;7) (7pter-7p14::1p36-1qter;

1pter-1p36::7p14-7qter)

t(14;20) (20qter-20q11::14p13-14qter;

20pter-20q11::14p13-14pter)

Fig. 1.—A clone cell from ataxia telangiectasia heterozygote, strain ATH4BI : 1

FIG. 2.—A clone cell from ataxia telangiectasia heterozygote, strain ATH1BI : 1

*Transformation with SV40 virus*

The rates of transformation of the fibroblast lines by SV40 virus are compared with chromosome damage levels in Table II. There is no obvious correlation between the level of chromosome damage present in a line of fibroblasts and the transformation rate found for that line.

1. Elevated rates were found for cells obtained from both patients with Fanconi's anaemia, but not for those from either of the patients with Bloom's syndrome.

2. Transformation rates for fibroblasts from the neurofibromatosis family showed a complex picture. None of the "non-involved" members showed increased rates, but of the 3 "involved" members 2 had normal rates and for the other patient, of 3 strains of cells studied, 1 had a normal rate and 2 had raised rates, one very considerably. This result was repeatable over several experiments and borne out in the "T" antigen study.

3. The heterozygotes for Fanconi's anaemia showed 1 out of 2 with a raised transformation rate, and of the non-clonal A-T heterozygote strains, one showed a transformation rate more than 3 times the average for normal cells (0·7 foci/5 $\times$ $10^4$ infected cells).

4. Rates for strains of fibroblasts carrying clones in high proportions are also shown in Table II. All 3 strains of A-T cells lay within the normal range as had been found for non-clonal A-T cells (Webb *et al.*, 1977). Of the 4 strains of cells from A-T heterozygotes which carried marked translocation clones, 2 had raised rates and 2 did not. Those with normal rates were both from the same patient, whereas those with increased rates were from 2 different patients.

TABLE II.—*The SV40 Transformation Rates of Fibroblasts from Individuals with Different Susceptibilities to Cancer*

| Cell line | % Clone | % Normal cells | % Gaps and breaks | Diagnosis | Transformation rate* | % Cells +ve for T antigen 72 h after infection |
|---|---|---|---|---|---|---|
| CON1 | — | 97 | 3 | Normal | 0·4 | — |
| CON2 | — | 90 | 7 | Normal | 0·3 | — |
| CON3 | — | 90 | 7 | Normal | 0·8 | — |
| CON4 | — | 97 | 3 | Normal | 0·5 | — |
| CON5 | — | 100 | 0 | Normal | 0·8 | 1·0 |
| CON6 | — | 100 | 0 | Normal | 1·2 | 2·0 |
| FA1 | — | 57 | 30 | Fanconi | 6·7±0·7 | 11·0 |
| FA2 | — | 75 | 16 | Fanconi | 13·7±4·8 | — |
| BL1 | — | 70 | 13 | Bloom | 0·7 | — |
| BL2 | — | 73 | 0 | Bloom | 2·0 | — |
| NF1 : 1 | 12 | 83 | 3 | Neurofibromatosis involved | 12·3±1·5 | 17·0 |
| NF1 : 2 | — | 93 | 3 | Neurofibromatosis involved | 4·3±1·2 | — |
| NF1 : 3 | — | 77 | 3 | Neurofibromatosis involved | 0·8±0·4 | — |
| NF2 | — | 90 | 7 | Neurofibromatosis involved | 1·0 | 1·5 |
| NF3 | — | 97 | 3 | Neurofibromatosis involved | 1·0 | — |
| NF4 | — | — | — | Uninvolved | 1·4 | — |
| NF5 : 1 | — | — | — | Uninvolved | 0·6 | — |
| NF5 : 2 | — | — | — | Uninvolved | 0·9 | 1·5 |
| FAH1 | — | 83 | 3 | Fanconi heterozygote | 4·0 | — |
| FAH2 | — | 97 | 3 | Fanconi heterozygote | 0·0 | — |
| ATH1BI : 2 | 13 | 77 | 0 | A-T heterozygote | 1·0 | — |
| ATH3BI : 1 | — | 93 | 3 | A-T heterozygote | 0·3 | — |
| ATH3BI : 2 | — | 83 | 3 | A-T heterozygote | 0·7 | — |
| ATH4BI : 2 | — | 90 | 7 | A-T heterozygote | 3·2 | — |
| ATH1BI : 1 | 74 | 17 | 8 | A-T heterozygote | 0·9 | — |
| ATH2BI | 97 | 3 | 0 | A-T heterozygote | 5·1±0·4 | — |
| ATH4BI : 1 | 33 | 60 | 0 | A-T heterozygote | 10·1±2·0 | 10·0 |
| AT8BI | 80 | 10 | 11 | A-T | 0·2 | — |
| AT9BI : 3 | 50 | 6 | 6 | A-T | 0·4 | — |
| AT9BI : 4 | 41 | 6 | 6 | A-T | 0·5 | — |

* Foci per 5 × $10^4$ cells plated out.

A direct comparison between A-T heterozygote strains ATH4BI : 1 and ATH1BI : 1 shows that both carry translocated clones (Fig. 1 and 2) but the transformation rate of ATH4BI:1 is 10·1±2·0 and that of ATH1BI : 1 is 0·9.

*SV40 T antigen*

In every case studied, a strong correlation was found between the percentage of cells which are positive for SV40 T antigen at 72 h after infection and the number of transformed colonies detected after 6 weeks of further incubation (Table II) (Aaronson and Todaro, 1968).

*Infection with SV40 DNA*

When the A-T heterozygote strain ATH4BI : 1 was infected with SV40 DNA instead of whole virus, the rate of transformation was no longer significantly greater than for the control cells (Table III). This is in agreement with the results obtained for a study of Fanconi fibroblasts, where the transformation level with SV40 DNA was also found to be reduced to that of normal cells (Aaronson, 1970). The level of chromosome damage, and the presence of SV40 T antigen in the cells infected with SV40 DNA, demonstrates interaction between virus DNA and the cell genome (Table III).

### DISCUSSION

A comparison has been made between cells derived from patients who have differing susceptibilities to malignant disease, both with respect to their chromo-

TABLE III.—*Chromosome Damage, Transformation Rate and T Antigen after Infection of 2 Fibroblast Strains with SV40 DNA*

| Cell line | No. of cells | Gaps and breaks before SV40 DNA infection | Gaps and breaks after SV40 DNA infection | Rings, dicentrics and fragments after SV40 DNA infection‡ | Transforma- rate* | % Cells +ve for T antigen 72 h after infection |
|---|---|---|---|---|---|---|
| CON 1 (normal) | 30 | 1 | 8 | 2 | 0·6 | 1·3 |
| † ATH4BI : 1 (A-T heterozygote) | 30 | 0 | 1 | 2 | 1·0 | 1·0 |

* Foci/5 × $10^4$ infected cells.
† A further endoreduplicated cell was observed with 2 Q-R.
‡ No rings, dicentrics or fragments were detected before infection.

some complements and their susceptibility to transformation by SV40 virus. Despite confirmation of earlier findings that FA fibroblasts have an increased transformation rate with SV40 virus (Todaro *et al.*, 1966) the relationship between the chromosome breakage syndromes, their increased susceptibility to malignant disease and their transformation rates with SV40 virus is further complicated by the finding that Bloom's syndrome fibroblasts, like those from A-T, are not unusually susceptible to transformation. Bloom's syndrome lymphocytes have been found to have a high frequency of sister chromatid exchanges (Chaganti, Schonberg and German, 1974) and to have slow DNA chain growth (Hand and German, 1975) but the basic defect has not yet been identified.

Deficiencies in DNA repair can be linked to the subsequent production of chromosome aberrations (Bender, Griggs and Bedford, 1974) but it has not been established whether the high SV40 transformation rates shown by FA cells are due to the reported DNA repair deficiency (Poon, O'Brien and Parker, 1974; Sasaki, 1975) or whether there is an earlier rate-limiting step. Virus penetration and uncoating have been suggested (Aaronson, 1970). Our finding that a fibroblast strain derived from an A-T heterozygote, although showing a high transformation rate after infection with SV40 virus, has a rate comparable to that of normal cells after infection with SV40 DNA lends support to these suggestions which were inferred from a study on Fanconi fibroblasts (Aaronson, 1970).

Of the 2 cell types with greatly increased susceptibilities to transformation, one was an A-T heterozygote strain carrying a balanced translocation clone, and the other was derived from a patient with neurofibromatosis, where 7/60 cells lacked a chromosome from the F group. The presence of other balanced translocations in A-T or A-T heterozygote cells did not cause raised transformation rates, so the presence of a clone *per se* does not appear to predispose the cell line to transformation, although it is possible to envisage that specific translocations may exert an effect. Potter and Potter (1975) found certain trisomic cells to be susceptible to SV40 transformation while others were not.

Although a high degree of heterogeneity has been observed in A-T families (Hoar and Sargent, 1976) it is difficult to visualize a system which confers increased transformation sensitivity upon the heterozygote (and then only in some strains) but not upon the homozygote. Until more studies have been undertaken to elucidate the status of the heterozygotes, we cannot assume the transformation sensitivity of cell strain ATH4BI : 1 to be a direct consequence of this genotype.

The variability of transformation rates found for different strains, although reproducible in themselves, reflect the heterogeneity of the families.

Although A-T heterozygotes have been shown statistically to be more susceptible to cancer than normal individuals (Swift *et al.*, 1975), apart from the tendency to fibroblast clone formation found here, no abnormalities have been reported at the cellular level. Despite the differences between the properties of cultured fibroblasts from FA patients and strain ATH4BI : 1, the increased transformation sensitivity shown by the A-T heterozygote strain follows the same pattern as that shown by FA fibroblasts, in that it disappears when SV40 DNA is used as the transforming agent.

The group of patients whose fibroblasts showed the greatest variation in their susceptibility to transformation by SV40 were those from the neurofibromatosis family. All the "non-involved" members, as expected, had rates lying within normal range, but those from the involved members varied considerably and care was taken to ensure that the differences were reproducible.

The striking increase in transformation level shown by NF1 : 1, was confirmed by the T antigen production of these cells at 72 h after infection. Even this result was not consistent however, for of the 3 involved members, only patient NF1 had raised levels, and then only in 2 of the 3 lines studied. The fact that different strains from one patient did not always show consistent susceptibility gives some measure of the complexity of the mechanism.

We would like to thank Professor J. Edwards, Dr J. Insley and Mr F. Court for biopsy material, and Miss A. Middleton for technical assistance. This work was supported by the Cancer Research Campaign.

### REFERENCES

AARONSON, S. A. (1970) Susceptibility of Human Cell Strains to Transformation by Simian Virus 40 and Simian Virus 40 Deoxyribonucleic Acid. *J. Virol.*, **6**, 470.

AARONSON, S. A. & TODARO, G. J. (1968) SV40 "T" Antigen and Transformation in Human Fibroblast Cell Strains. *Virology*, **36**, 254.

BENDER, M. A., GRIGGS, H. G. & BEDFORD, J. S. (1974) Mechanisms of Chromosomal Aberration Production. III. Chemicals and Ionising Radiation. *Mut. Res.*, **23**, 197.

CASPERSSON, T., ZECH, L. & JOHANSSON, C. (1970) Differential Binding of Alkylating Fluorochromes in Human Chromosomes. *Expl Cell Res.*, **60**, 315.

CHAGANTI, R. S. K., SCHONBERG, S. & GERMAN, J. (1974) A Manyfold Increase in Sister Chromatid Exchanges in Bloom's Syndrome Lymphocytes. *Proc. natn. Acad. Sci. USA*, **71**, 4508.

COHEN, M. M., SHAHAM, M., DAGAN, J., SHMUELI, E. & KOHN, G. (1975) Cytogenetic Investigations in Families with Ataxia Telangiectasia. *Cytogen. Cell Gen.*, **15**, 338.

GERMAN, J. & PUGLIATTI CRIPPA, L. (1966) Chromosome Breakage in Diploid Cell Lines from Bloom's Syndrome and Fanconi's Anaemia. *Annls Genet.*, **9**, 143.

GRAHAM, F. L., ABRAHAMS, P. J., MULDER, C., HEIJNEKER, H. L., WARNAAR, S. O., DE VRIES, F. A. J., FIERS, W. & VAN DER EB, A. J. (1974) Studies on *In Vitro* Transformation by DNA and DNA Fragments of Human Adenoviruses and Simian Virus 40 *Cold Spring Harbor Symp. quant. Biol.* **39**, p. 637.

HAND, R. & GERMAN, J. (1975) A Retarded Rate of DNA Chain Growth in Bloom's Syndrome. *Proc. natn. Acad. Sci.*, **72**, 758.

HARNDEN, D. G. (1974) Skin Culture and Solid Tumour Technique. In *Human Chromosome Methodology*. Ed. J. J. Yunis. London and New York: Academic Press. p. 167.

HARNDEN, D. G., BENN, P. A., OXFORD, J. M., TAYLOR, A. M. R. & WEBB, T. (1976) Cytogenetically Marked Clones in Human Fibroblasts Cultured from Normal Subjects. *Somatic Cell Gen.*, **2**, 55.

HIRT, B. (1967) Selective Extraction of Polyoma DNA from Infected Mouse Cell Cultures. *J. mol. Biol.*, **26**, 365.

HOAR, D. I. & SARGENT, P. (1976) Chemical Mutagen Hypersensitivity in Ataxia Telangiectasia. *Nature, Lond.*, **261**, 590.

KERSEY, J. H., GATTI, R. A., GOOD, R. A., AARONSON, S. A. & TODARO, G. J. (1972) Susceptibility of Cells from Patients with Primary Immunodeficiency Diseases to Transformation by Simian Virus 40. *Proc. natn. Acad. Sci. USA*, **69**, 980.

MUKERJEE, D., BOWEN, J. H. & ANDERSON, D. E. (1970) Simian Papova Virus 40 Transformation of Cells from Cancer Patient with XY/XXY Mosaic Klinefelter's Syndrome. *Cancer Res.*, **30**, 1769.

MUKERJEE, D., BOWEN, J. M., TRUJILLO, J. M. & CORK, A. (1972) Increased Susceptibility of Cells from Cancer Patients with XY-gonadal Dysgenesis to Simian Papova Virus 40 Transformation. *Cancer Res.*, **32**, 1518.

POON, P. K., O'BRIEN, R. L. & PARKER, J. W. (1974) Defective DNA Repair in Fanconi's Anaemia. *Nature, Lond.*, **250**, 223.

POTTER, A. M. & POTTER, C. W. (1975) Transformation of Human Cells by SV40 Virus. *Br. J. Cancer*, **31**, 348.

SAMBROOK, J., WESTPHAL, H., SRINIVASAN, P. R. & DULBECCO, R. (1968). The Integrated State of Viral DNA in SV40-transformed Cells. *Proc. natn. Acad. Sci. USA*, **60**, 1288.

SASAKI, M. S. (1975) Is Fanconi's Anaemia Defective in a Process Essential to the Repair of DNA Cross Links. *Nature, Lond.* **257**, 501.

SEABRIGHT, M. (1971) A Rapid Banding Technique for Human Chromosomes. *Lancet*, ii, 971.

SWIFT, M. (1973) Fanconi's Anaemia in the Genetics of Neoplasia. *Nature, Lond.*, **230**, 370.

SWIFT, M., SHOLMAN, L., PERRY, M. & CHASE, C. (1975) Malignant Neoplasms in the Families of Patients with Ataxia Telangiectasia. *Cancer Res.*, **36**, 209.

TODARO, G. J., GREEN, H. & SWIFT, M. R. (1966) Susceptibility of Human Diploid Fibroblast Strains to Transformation by SV40 Virus. *Science, N.Y.*, **153**, 1252.

TODARO, G. J. & MARTIN, G. M. (1967) Increased Susceptibility of Down's Syndrome Fibroblasts to Transformation by SV40. *Proc. Soc. exp. Biol. Med.*, **124**, 1232.

WEBB, T. & HARNDEN, D. G. (1976) The Transformation by Simian Virus 40 of Cells from Patients with Mucopolysaccharidosis and from Normal Controls. *Cancer Res.*, **36**, 298.

WEBB, T., HARNDEN, D. G. & HARDING, M. (1977) The Chromosome Analysis and Susceptibility to Transformation by SV40 of Fibroblasts from Ataxia Telangiectasia. *Cancer Res.*, **37**, 997.